# Unmet needs in EGFR exon 20 insertion mutations in Central and Eastern Europe: reimbursement, diagnostic procedures, and treatment availability

**DOI:** 10.1186/s12919-023-00287-6

**Published:** 2024-01-18

**Authors:** Maximilian J. Hochmair, Mojca Unk, Jelena Spasic, Timur Cerić, Assia Konsoulova, Mircea Dediu, Krisztina Bogos, Alinta Hegmane, Kersti Oselin, Marko Stojiljkovic, Tina Roblek, Marko Jakopovic

**Affiliations:** 1 Department of Respiratory and Critical Care Medicine, Karl Landsteiner Institute of Lung Research and Pulmonary Oncology, Klinik Floridsdorf, Vienna, Austria; 2 Institute of Oncology Ljubljana, Zaloška Cesta 2, Ljubljana, Slovenia; 3 Institute for Oncology and Radiology of Serbia, Pasterova 14, Belgrade, Serbia; 4 University Clinical Center Sarajevo, Bolnička 25, Sarajevo, Bosnia and Herzegovina; 5 National Oncology Hospital, “Plovdivsko Pole” 6, Sofia, 1756 Bulgaria; 6 Sanador Oncology Center Bucharest, Strada Sevastopol 5, Bucharest, Romania; 7 National Koranyi Institute for Pulmonology, Korányi Frigyes út 1, Budapest, Hungary; 8 Riga East University Hospital, Oncology Center of Latvia, Hipokrāta iela 4, Rīga, Latvia; 9 North Estonia Medical Centre, J. Sütiste tee 19, Tallinn, Estonia; 10 Takeda d.o.o., Bulevar Milutina Milankovića 11a, Belgrade, Serbia; 11 Takeda Pharmaceuticals d.o.o., Bleiweisova cesta 30, Ljubljana, Slovenia; 12 Zagreb Medical School, University Clinical Hospital Center Zagreb, Jordanovac 104, Zagreb, Croatia

**Keywords:** *EGFR* exon 20 insertion mutations, NSCLC treatment, Diagnostic challenges, Targeted therapies, Next-generation sequencing, Healthcare disparities in Central and Eastern Europe

## Abstract

Lung cancer remains the leading cause of cancer-related deaths in Europe, with non-small cell lung cancer (NSCLC) accounting for approximately 85% of cases. NSCLC is a heterogeneous disease encompassing various oncogenic alterations. Among them, *EGFR* exon 20 insertion mutations, constituting 0.3–2.2% of NSCLC cases, rank as the third most common *EGFR* alteration after exon 19 deletions and the L858R point mutation in exon 21, also known as “typical” *EGFR* alterations. Recent advancements in understanding the molecular pathogenesis of NSCLC have led to significant breakthroughs in targeted therapies, revolutionizing treatment options for patients with specific genetic alterations.

This article presents the outcomes of a Virtual Meeting conducted on the online platform (provided Within3©) from September 19 to October 30, 2022. The meeting focused on addressing the challenges in the diagnosis and treatment of NSCLC patients with *EGFR* exon 20 insertion mutations. The participants consisted of healthcare professionals from ten Central and Eastern European countries who shared their experiences and opinions on various aspects, including epidemiology, treatment options, and diagnostic approaches employed in their respective healthcare institutions. The discussions were facilitated through open-ended and multiple-choice questions.

The primary objective of this article is to provide an overview of the identified challenges associated with the diagnosis and treatment of this heterogeneous disease, based on the assessments of the meeting participants. Among the major emerging challenges discussed, the reimbursement issues concerning next-generation sequencing (NGS), a recommended method in NSCLC molecular diagnosis, and the availability of approved targeted treatments to enhance patient outcomes were of paramount importance. Furthermore, fostering community awareness of lung cancer and promoting harmonized lung cancer care were identified as areas deserving greater attention. Notably, the rapidly evolving treatment landscape, particularly with NGS for NSCLC patients with genomic alterations like *EGFR*, *ALK*, *RET*, *MET*, *NTRK*, and *ROS1*, necessitates prioritizing the development of new drugs, even for the relatively smaller subgroup with exon 20 insertion mutations.

## Introduction

Globally, lung cancer is the second most diagnosed cancer with over 2.2 million new cases (11.4% of all cases) for both sexes combined and the leading cause of cancer-related deaths (18.0% of all sites) with nearly 1.8 million new deaths, in 2020 [1]. In Europe, lung cancer was the third most common cancer with 470,000 (12.0%) new cases following breast and colorectal cancer, and the most frequent cause of cancer mortality with an estimated 388,000 deaths (one-fifth of the total) in 2018 [2].

Non-small cell lung cancer (NSCLC) comprises approximately 85% of all lung cancer cases [3]. NSCLC is a heterogenous disease, characterized by numerous oncogenic alterations. One of dominant alterations in NSCLC occurs in epidermal growth factor receptor (*EGFR*) [4]. The *EGFR* alterations have been reported to represent approximately 14% of all cases of NSCLC in European Countries [4]. The most frequent alterations in the *EGFR* gene include deletions in exon 19 and the L858R point mutation in exon 21, which are also known as “typical” *EGFR* alterations [5]. They constitute approximately 85% of *EGFR* alterations, while the remaining 10–15% are made up of atypical *EGFR* alterations, including exon 18 and exon 20 alterations [4]. According to a meta-analysis based on nine studies, conducted by Van Sanden et al. in 2022, the frequency of exon 20 insertion alterations ranged 2.5–23.1% within *EGFR* positive patients with NSCLC and 0.3–2.2% amongst a general NSCLC population [5]. Furthermore, *EGFR* alterations are more common among female and never-smoker patients with adenocarcinoma [6–8].

Polymerase chain reaction (PCR) and next-generation sequencing (NGS) are the methods used for the detection of *EGFR* exon 20 insertion mutations, however, Bauml et al. showed that PCR would be expected to miss half of the NGS-identified mutations [9]. Ou et al. also analyzed the detectability of six different commercially available and widely used PCR kits for *EGFR* exon 20 insertion variants that were identified using NGS or other conventional sequencing testing [10]. The study revealed that more than 40% of patients with *EGFR* exon 20 insertion variants would have been missed by the PCR tests evaluated, and the authors pointed out NGS-based genetic testing could be preferable than standard PCR assays. As the number of approved biomarkers for actionable targets increases, a multi-gene approach is recommended, preferably by NGS, rather than single-gene *EGFR* testing in several guidelines [11, 12].

The increasing molecular understanding of NSCLC has led to dramatic developments in the treatment options for patients with tumors, harboring genetic alterations. Tyrosine kinase inhibitors (TKIs) are nowadays the recommended first-line treatment for patients with classical *EGFR* alterations. However, being a heterogeneous molecular subgroup, patients with *EGFR* exon 20 insertion mutations are difficult to treat, as most TKIs generally have limited efficacy [13]. Certain *EGFR* exon 20 insertion mutations, such as *S768_D770dup* and *H773L/V774 M*, have shown promising responses to osimertinib, as demonstrated in NSCLC patients. This underscores the potential sensitivity of specific *EGFR* exon 20 insertion mutations to osimertinib therapy, emphasizing the need for further research in understanding their varied responses [14]. In May 2021, amivantamab, a human bispecific antibody for *EGFR* and mesenchymal-epithelial transition (MET) receptor, was the first drug approved by US Food and Drug Administration (FDA) for patients with advanced or metastatic NSCLC with exon 20 insertion mutations following progression after platinum-based chemotherapy [15]. In December 2021, the European Medicines Agency granted marketing authorization for amivantamab for the same indication [16]. Subsequently, the CHRYSALIS study, a phase I trial, explored amivantamab’s efficacy and safety in this patient population, revealing a robust 40% overall response rate, including three complete responses, with a median duration of response of 11.1 months. The study also reported a median progression-free survival of 8.3 months. Adverse events, such as rash and infusion-related reactions, were common but manageable, with treatment-related dose reductions and discontinuations reported in 13% and 4% of patients, respectively. These comprehensive findings underscore amivantamab’s potential as a valuable therapeutic option for patients with *EGFR* exon 20 insertion mutations after platinum-based chemotherapy [17]. Additionally, a phase 3 international randomized trial, known as the PAPILLON study, further supports the efficacy of amivantamab in treating advanced non-small-cell lung cancer (NSCLC) with *EGFR* exon 20 insertions. The trial compared intravenous amivantamab plus chemotherapy (amivantamab-chemotherapy) to chemotherapy alone in patients who had not received previous systemic therapy. Results demonstrated a significant improvement in progression-free survival in the amivantamab-chemotherapy group (median, 11.4 months) compared to the chemotherapy group (median, 6.7 months), with a hazard ratio for disease progression or death of 0.40 (95% CI, 0.30 to 0.53; *P* < 0.001). At 18 months, progression-free survival rates were 31% and 3% in the amivantamab-chemotherapy and chemotherapy groups, respectively, further supporting the favorable outcomes of amivantamab-based therapy [18]. Another drug, mobocertinib, a selective oral TKI targeting *EGFR* and *HER2* exon 20 insertion mutations, also received accelerated approval for this indication from US FDA in September 2021 [19]. A subsequent open-label, phase 1/2 nonrandomized clinical trial investigated mobocertinib’s efficacy and safety in patients with previously treated *EGFR* exon 20 insertion-positive metastatic non-small cell lung cancer (mNSCLC). The study, including platinum-pretreated patients and an extension cohort, demonstrated that mobocertinib, administered at a dose of 160 mg once daily, achieved a confirmed objective response rate (ORR) of 28% by independent review committee (IRC) assessment and 35% by investigator assessment in the platinum-pretreated cohort. Diverse EGFR exon20 insertions variants were analyzed, revealing a higher IRC-assessed confirmed ORR (32%) in patients with *ASV*, *SVD*, or *NPH* variants compared to those with less frequent variants (25%), emphasizing the influence of specific mutation subtypes on treatment outcomes. The response rates were similar regardless of whether insertion mutations occurred in near-loop or far-loop positions. The drug showed a manageable safety profile, with diarrhea and rash being the most common treatment-related adverse events. These results suggest that mobocertinib could be a promising therapeutic option for patients with *EGFR* exon 20 insertion-positive mNSCLC after prior platinum-based treatment [20].

The aim of this report is to provide an overview of experts’ experiences in the treatment and diagnostic challenges related to *EGFR* exon 20 insertion mutations in NSCLC, provided in based on the insights collected in the Virtual Meeting held from September 19 to October 30, 2022. The participating healthcare professionals, who practice in ten Central and Eastern European Countries (Austria, Bosnia and Herzegovina, Bulgaria, Croatia, Estonia, Hungary, Latvia, Romania, Serbia, and Slovenia), discussed topics such as diagnostic methods, epidemiology, and treatment options for patients with NSCLC, including those with *EGFR* exon 20 insertion mutations. These topics were covered through open and/or multiple-choice questions.

## Diagnostic patterns of patients

According to the experts participating in the study, the annual incidence of NSCLC patients diagnosed with *EGFR* exon 20 insertion is estimated to be approximately 1–2 cases in Bosnia and Herzegovina, Bulgaria, and Estonia. These figures represent an underestimate of the actual incidence, as they result from a deficiency in systematic molecular studies. However, a Slovenian specialist reported that their country experiences 5 cases per year. The highest annual incidence, as evaluated by the participants, was observed in Croatia, with approximately 5–10 cases annually.

Regarding *EGFR* alteration testing, the approaches vary among countries. Half of the experts stated that *EGFR* testing is reflexive for all non-squamous NSCLC patients at the time of diagnosis. However, in Estonia, Hungary, Latvia, and Serbia, testing is restricted to advanced or metastatic disease due to reimbursement limitations. In terms of non-smoker patients with squamous cell carcinoma, only a Romanian expert mentioned routine testing, while in Croatia, Serbia, and Slovenia, it is performed upon request. Additionally, screening for the *T790M* mutation is conducted at disease progression after the use of first/second generation TKIs or third generation TKI. None of the participating countries currently have registries specifically including patients with *EGFR* exon 20 insertion mutations. However, the majority of experts expressed interest in future collaborations to establish such registries. While Austria has a lung cancer registry for diagnosis and treatment, it does not specifically focus on patients with *EGFR* alterations [21].

Among the participating experts, only those from Slovenia, Bulgaria, and Hungary indicated the presence of local diagnostic guidelines in their respective countries. However, the remaining participants (6) emphasized the absence of guidelines in their countries. The majority of the experts reported a lack of local diagnostic guidelines for NSCLC. As a result, a few of the participating specialists are following the international guidelines provided by ESMO (European Society for Medical Oncology). Furthermore, an expert from Bosnia and Herzegovina highlighted that their local guidelines are currently being developed. As for the diagnostic periods, from a general practitioner referral until an oncologist’s exam might often take 1–2 weeks. In terms of diagnostic timelines, the period between a general practitioner referral and an oncologist’s examination often ranges from 1–2 weeks. The average time required for scheduling diagnostic procedures and histological sampling may vary, with some cases taking less than a week and others exceeding 1 month. However, most experts shared that the process typically takes up to 2 weeks, while specialists from Serbia and Romania mentioned that it can take up to 4 weeks in their respective countries. Only a participant from Hungary mentioned that this process can extend beyond a month in their institution.

When considering the average time needed to obtain results of predictive biomarker testing, the majority of experts stated that it usually takes 7–10 working days. However, a Hungarian specialist indicated that in their institution it can take up to 14 days. Additionally, an expert from Latvia stated that in their country, results can be obtained within 5–7 working days. Most of the experts estimated the average time to obtain results of predictive biomarkers as 5–10 working days.

According to the participating experts, the time from the final pathohistological diagnosis until the treatment decision by oncologists or multidisciplinary teams is mostly up to 2 weeks. However, a Bulgarian specialist stated that this process can take more than 1 month. Regarding the time necessary from the treatment decision until treatment initiation for first-line treatments, most participating experts mentioned that it usually takes up to 2 weeks. However, specialists practicing in Serbia and Romania indicated that this process could last for up to 3–4 weeks. A Serbian expert emphasized that this process could be shortened if the institution had larger capacities for patients and more available staff.

In general, the assessment of patients showed that the shortest periods were for the treatment decision by an oncologist/multidisciplinary team (MDT) and first-line treatment initiation, both typically taking less than 1 week. Nevertheless, all experts agreed that none or very few patients (fewer than 10%) experienced deterioration or were lost to follow-up during the diagnostic periods.

All the provided information was shared by participating healthcare professionals and reflects their personal experiences acquired while practicing in institutions across Central and Eastern Europe. It should be noted that their estimations have not been validated by hospitals’ medical records.

PCR, fast PCR, reverse transcription-PCR, and NGS were the methods to detect *EGFR* alterations. When the tissue sample was of suboptimal size or quality, re-biopsy or in some cases, circulating tumor DNA analysis using liquid biopsy were the options for search of *EGFR* alteration in all countries. Over the course of the disease, in case of progression, almost all experts indicated Cobas based liquid biopsy for follow-up of patients with *EGFR* positive tumors. The quality assurance systems applied in each country for predictive biomarker assessment in patients with NSCLC were different. Generally, laboratories/pathology centers had internal procedures and certifications, and each country had its own system and variable external quality assessments for quality assurance. It was pointed out that largely the centers were in concordance and the procedures were well established, hence, there was no heterogeneity among them, mostly due to the small number of centers having biomarker testing and reimbursement systems throughout the country.

**Fig. 1:**
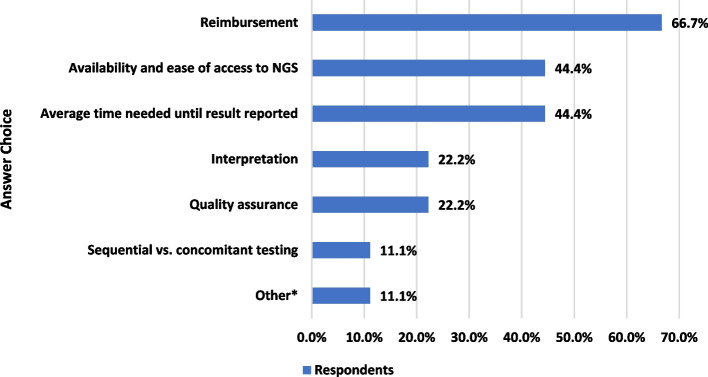
Identified challenges related to predictive biomarker assessments in patients with NSCLC, according to participants in the Virtual Meeting. Answers were provided by 9 participants via a multiple-choice question. * Specific determination of exon 19 and exon 21 mutations only

The challenges related to predictive biomarker assessments in patients with NSCLC are shown in Fig. 1. Two-thirds of the participating experts emphasized the reimbursement issues as the most frequent challenge. Although PCR and immunohistochemistry reimbursements were fully or partially supported by pharmaceutical companies in many countries, patients needed to pay for NGS, which was fully reimbursed only in three (Slovenia, Hungary and Latvia) out of nine participants’ home countries. Furthermore, due to the drug reimbursement restrictions after the test, recommendation of NGS testing at the patients’ out-of-pocket expense is rare. Therefore, NGS could not be routine at this point, although concomitant evaluation of predictive biomarkers in patients with NSCLC was accepted as a rule and half of the participants indicated that testing was reflex. The participants mostly suggested that NGS reimbursement fully/partially or collaboration between pharmaceutical companies and laboratories could be established in order to facilitate access and development of NGS diagnosis in Eastern European countries. Furthermore, specialists from Romania, Serbia, Bosnia and Herzegovina, and Estonia unanimously agreed that NGS diagnostics are not reimbursed by local health insurances. They also mentioned that it is unlikely to change within the next 3 years.

Overall, no significant challenges were mentioned regarding the interpretation of predictive biomarker assessment in patients with NSCLC. However, nearly half of the participants highlighted the importance of a molecular tumor board for reviewing results, especially in cases involving NGS, which was not routinely performed due to reimbursement constraints. In certain countries, testing had to be initiated by a multidisciplinary team, potentially causing delays in patient management.

Community awareness about lung cancer was identified as a crucial issue and improving the time from symptom onset to presentation to general practitioners and pulmonologists was deemed necessary to expedite diagnosis and treatment. Moreover, participants suggested that education and awareness campaigns targeting physicians could prove beneficial in reducing diagnosis time, ensuring appropriate timing of tests, and addressing reimbursement challenges.

## Treatment options for patients with *EGFR* exon 20 insertion mutations

In general, all patients with identified *EGFR* exon 20 mutations were treated with first-line therapy, but the primary determining criteria for the treatment were mainly performance status and comorbidities. The percentage of patients treated with the second-line therapy varied between 50 to 80% and it was mentioned that 10% of the patients could receive three or more lines of therapy. Platinum-based chemotherapy and/or immune checkpoint inhibitors (ICIs) were preferred by more than half of the participating experts in the first-line treatment, and the remaining ones mentioned afatinib as the first choice. Experts mentioned mobocertinib or amivantamab preferred as options in the second-line treatment, but chemotherapy/immunotherapy was said to be administered in real-life settings due to the availability of the aforementioned treatments. Therefore, if chemotherapy was used in the first-line treatment, docetaxel +/- nintedanib or mobocertinib/afatinib were listed as given second-line treatments. Besides, platinum-based chemotherapy was administered in the second-line treatment to patients with *EGFR* exon 20 insertion mutations after being treated with afatinib.

Preferred choice of treatments in the third line varied depending on the performance status, availability or previous use of drugs. Mobocertinib, amivantamab, and poziotinib were mentioned in this line. Besides, immunotherapy and chemotherapy or the combination of these treatments might also be preferred. Lastly, regarding the later lines of treatment, best supportive care was the most frequent answer. In some cases, chemotherapy could also be carried out. Yet, the clinical/biological profile of the patient was another determining factor in the choice of treatment.

In the current treatment landscape, availability of targeted treatments for patients harboring *EGFR* exon 20 insertion mutations was assumed as the most critical unmet medical need to addressed. Croatian and Slovenian specialists emphasized that drugs such as mobocertinib and amivantamab are not reimbursed, which affects treatment outcomes and delays NGS testing. Furthermore, the lack of reimbursement for mobocertinib in Croatia was mentioned as an obstacle to improving treatment outcomes for patients. Resolving the reimbursement issue for these drugs could also expedite the reimbursement process for NGS testing.

## Conclusion

The improvements in understanding the molecular pathogenesis of NSCLC have revealed a variety of oncogenic changes and in the most recent decades targeted therapies revolutionized the treatment landscape in lung cancer. The Virtual Meeting provided insights into identified challenges of diagnosis and treatment of patients with NSCLC, harboring *EGFR* exon 20 insertion mutations based on the view of experts from Austria, Bosnia and Herzegovina, Bulgaria, Croatia, Estonia, Hungary, Latvia, Romania, Serbia, and Slovenia. There is a high diversity in the economic status of the participants’ home countries, resulting in differences in reimbursement and availability of diagnostic procedures and treatment options. The biggest identified unmet needs were NGS reimbursement, which was recognized by most participating experts, except for specialists from Slovenia, Hungary, and Latvia, where NGS is already reimbursed by their health insurance. A Croatian specialist stressed the lack of reimbursement for certain drugs, such as amivantamab and mobocertinib, as an important issue. In terms of timepoints in diagnostic procedures, only in a Hungarian healthcare institution does it take more than 1 month from scheduling a diagnostic procedure to obtaining the sample, which is longer compared to other countries. Additionally, as stated by a participating expert from Bulgaria, the period from the final pathohistological diagnosis to the treatment decision made by oncologists or multidisciplinary teams can exceed 1 month in their country. Generally, expanding the availability of approved targeted treatments and diagnostic procedures was recognized as a crucial step towards improving therapy outcomes. Furthermore, it is suggested that prioritizing the awareness of the population about lung cancer, harmonizing lung cancer care, and making simultaneous testing available for all biomarkers could be beneficial.

## Data Availability

All the transcripts of the questions, insights and comments are available at Takeda doo and by the corresponding author.

## Abbreviations

*ALK*: Anaplastic Lymphoma Kinase
CI: Confidence Interval
*EGFR*: Epidermal Growth Factor Receptor
ESMO: European Society for Medical Oncology
FDA: Food and Drug Administration
ICIs: Immune Checkpoint Inhibitors
IRC: Independent Review Committee
mNSCLC: Metastatic Non-Small Cell Lung Cancer
MDT: Multidisciplinary Team
*MET*: Mesenchymal-Epithelial Transition
*NTRK*: Neurotrophic Tyrosine Receptor Kinase
NGS: Next-Generation Sequencing
NSCLC: Non-Small Cell Lung Cancer
ORR: Overall Response Rate
PCR: Polymerase Chain Reaction
*RET*: Rearranged During Transfection
*ROS1*: Receptor tyrosine kinase
*TKIs*: Tyrosine Kinase Inhibitors
